# Regulation effects of total flavonoids in *Morus alba* L. on hepatic cholesterol disorders in orotic acid induced NAFLD rats

**DOI:** 10.1186/s12906-020-03052-w

**Published:** 2020-08-17

**Authors:** Yucheng Hu, Jingqi Xu, Qian Chen, Mengyang Liu, Sijian Wang, Haiyang Yu, Yi Zhang, Tao Wang

**Affiliations:** 1 Institute of Traditional Chinese Medicine, Tianjin University of Traditional Chinese Medicine, Tianjin, 300193 China; 2 Tianjin State Key Laboratory of Modern Chinese Medicine, Tianjin University of Traditional Chinese Medicine, Tianjin, 300193 China

**Keywords:** Mulberry leaves flavonoids, Quercetin, Cholesterol metabolism, NAFLD

## Abstract

**Background:**

Mulberry leaves are the dried leaves of *Morus alba* L., flavonoids from mulberry leaves (MLF) has showed regulatory effect on abnormal lipid metabolism, but the regulatory mechanism of MLF on cholesterol metabolism is still missing. This study was designed to investigate the effect of MLF and its active metabolite quercetin on regulating cholesterol disorders.

**Methods:**

The mechanism of MLF on alleviating liver injury and regulating cholesterol was examined in dyslipidemic SD rats. The regulatory mechanism of quercetin for cholesterol disorders have also been detected through lipid laden HepG2 cell model.

**Results:**

Our results showed that MLF significantly inhibited lipid accumulation and alleviate hepatic injury in NAFLD rat model. The hepatic expression level of SREBP2, HMGCR and miR-33a were significantly down-regulated, while CYP7A1 was induced by MLF treatment. In vitro, Quercetin significantly decreased lipid accumulation in HepG2 cells. Mechanistically, quercetin could inhibit the mRNA and protein expression level of SREBP2 and HMGCR with or without LDL treatment. In addition, quercetin could also reduce the LXRβ while induced SR-BI mRNA expression.

**Conclusion:**

Our findings indicate that MLF and quercetin could reduce the excessive cholesterol accumulation in vivo and in vitro. These cholesterol-regulating phenomenon might attribute to its effect on down-regulating the expression of lipid-related markers such as SREBP2 and HMGCR, which may exert a protective role in the NAFLD treatment.

## Background

Nonalcoholic fatty liver disease (NAFLD) is a common chronic disease characterized by excessive accumulation of lipids in the liver other than alcohol intake [1]. Although NAFLD is not fatal, it is considered as main risk factors of liver cancer and cardiovascular disease (CVD), such as hypertension, arteriosclerosis and coronary heart disease [2]. At present, the correlation between NAFLD and CVD is still unclear, but the results indicate that lipid accumulation is considered as the important mediator between the two risks [3]. A previous meta-analysis showed that NAFLD patients had a 64% higher risk of fatal and/or non-fatal CVD events than non-NAFLD patients [3].

Elevated serum cholesterol and low-density lipoprotein-cholesterol (LDL-C) levels are the main triggers of NAFLD caused CVD [4, 5]. Under normal condition, synthesis of cholesterol is tightly regulated by sterol regulatory element binding protein 2 (SREBP2) and its down-stream cholesterol synthesis enzymes such as HMG-CoA reductase (HMGCR) [6, 7]. Nearly 60% synthesized cholesterol is transported from liver to plasma and plays an important role in keeping membrane structure and synthesis of hormones, and nearly 40% cholesterol is converted to bile acid by cholesterol-7α-hydroxylase (CYP7A1) [8]. Under the state of NAFLD, excessive lipid accumulation leads to steatosis in liver, and causes the up-regulation of SREBP2 and HMGCR, which results in cholesterol over-production [9]. At the same time, the increase of cholesterol content in the liver will reduce the conversion rate of bile acid and reduce cholesterol intake from the serum. These changes can further disrupt cholesterol homeostasis and increase the risk of CVD.

Natural products have been used to treat various kinds of diseases, owing to its anti-tumor, anti-inflammatory, antioxidant and hepatoprotective properties [10–12]. Due to the low toxicity and side effects of natural drugs, 40% of FDA-approved treatment are natural ingredients or derivatives [12, 13].

As a life style related disease, there is no specific medication to treat moderate NAFLD in clinic. Dietary supplements or complementary alternatives are becoming a choice for prevention and therapy of NAFLD [14]. And inhibition of NAFLD through the natural products have been extensively studied and reported [15].

Mulberry leaves are the dried leaves of *M. alba* L., which is used as the medicinal and edible plants in south-east Asia for a long period [16]. The chemical constituents of mulberry leaves contain flavonoids (quercetin and its homologue), alkaloids (deoxynojirimycin), sitosterols and polysaccharides. Within them, quercetin and its homologue are the major constituents and the content is more than 1% [17]. It has been reported that mulberry leaves have the pharmacological effect of attenuating hepatic steatosis and insulin resistance in high-fat diet-fed mice [18]. And its active compound quercetin could reduce postprandial blood glucose, lowering blood lipid, anti-inflammation and anti-tumor [19]. In addition, quercetin was also used to regulate hepatic lipid metabolism in traditional clinic [20]. Recently, it was reported that mulberry leaves extract showed significant effect on reducing plasma cholesterol level in rats induced by high-fat diet, but the mechanism of action is not clear [18].

In this study, we investigated the mechanism of the flavonoids of mulberry leaves (MLF) on cholesterol regulation in dyslipidemic rats. In addition, the role of quercetin, main metabolites of flavonoids in MLF, in cholesterol disorders was also determined in lipid laden HepG2 cell model, and the mechanism of its action was further explored.

## Methods

### Plant material

The mulberry leaves were collected from Dezhou county, Shandong province, China, and identified by Dr. Yi Zhang as leaves of *M. alba* L. The voucher specimen was deposited at the Academy of Traditional Chinese Medicine of Tianjin University of Traditional Chinese Medicine (TJUTCM; No.2013083101).

Methanol extract of the leaves of *M. alba* L. was provided by the Chinese Medicine Chemistry Laboratory of TJUTCM, stored at the room. In brief, the mulberry leaves were ground (500 g) and extracted with methanol (5 L) under reflux for two times. The combined extracting solution was concentrated in vacuum to afford a methanol extract. Quercetin and rutin content in methanol extract were 0.9 and 2.6% determined by HPLC method described previously [21]. Quercetin standard sample was obtained from Shanghai yuanye Bio-Technology Co., Ltd.

### Animals and treatment

Healthy male Sprague-Dawley (SD) rats (100–120 g, 4 weeks old) were purchased from Vital River Laboratory Animal Technology Co., Ltd., (Beijing, China). All rats had a free access to water and standard chow diets and were housed in cages with temperature-controlled (20–26 °C) for 12 h of light/dark cycles. After a week of adaptation, the rats were divided into six groups (*n* = 10 per group), including normal group (Normal: a balanced diet), control group (Control: 1% OA diets with 33% sucrose content), fenofibrate group (Feno: OA diets with oral administration of 50 mg kg^− 1^ d^− 1^), and methanol extract of mulberry leaves groups (MLF: OA diets with oral administration of MLF 200, 100 and 50 mg kg^− 1^ d^− 1^). The maximum oral administration doses for rat (200 mg kg^− 1^ d^− 1^) was translated from the traditional human daily dose for fatty liver disease and extraction yield rate. The test samples were dissolved in solution of 5% gum arabic and administered orally to rats via gavage. Orotic acid (OA) and uratan were obtained from Shanghai Future Industry Co, Ltd. and Shanghai Ekear Co, Ltd. respectively. All other reagents were purchased from Sigma-Aldrich except where indicated. All animal procedures were performed in compliance with the Guidance Suggestions for the Care and Use of Laboratory Animals issued by the Ministry of Science and Technology of China (2006) and Science and Technological Committee and the Animal Use and Care Committee of TJUTCM (No. 201810009).

### Determination of hepatic lipid content

The rats were fasted for 12 h at the end of the experiment. The rats were anesthetized by intraperitoneal injection of 20% uratan with a dose of 1 ml/100 g (body weight). After rats dissected, the blood was pumped out of the liver by D-Hanks perfusion through portal vein, and the liver was removed and rinsed with pre-cooled normal saline, and then the rats were sacrificed by cervical dislocation. Liver total lipid content was extracted as previously described with slight modifications [22]. The commercial assay kits were used to detect the contents of total cholesterol (TC) and liver triglyceride (TG) in the liver.

### Hepatic histological evaluation

The liver samples were fixed in 4% paraformaldehyde, dehydrated in ethanol gradient solutions and embedded in paraffin wax for hematoxylin-eosin (H&E) staining. The liver frozen sections were stained with Oil Red O as previously described [23]. And an Axio Imager D2 microscope (Zeiss, Oberkochen, Germany) was applied to take photographs for histological and morphological analysis. H&E-stained liver sections were assessed blindly for NAFLD activity score (NAS) and lobular inflammation [24, 25]. The number of fat drops and fat distribution in liver tissues and hepatocytes were observed. The following scoring criteria were used to score the steatosis of liver tissues. If the distribution of lipid droplets in hepatocytes is rare, it is denoted as 0. No more than 25% of the area will be counted as 1 point. No more than 50% is counted as 2 points, no more than 75% as 3 points, and almost all of the lipid droplets account for 4 points. The scoring criteria of hepatic lobular inflammation were as follows. If no necrosis is seen, it is denoted as 0. A small amount of inflammatory cell infiltration and necrosis was counted as 1 point. Moderate inflammatory cell infiltration was recorded as 2 points and massive necrosis was recorded as 3 points.

### Immunohistochemistry assay

To determine SREBP2, HMGCR and CYP7A1 expression, the liver sections were permeabilized with 0.5% (v/v) Triton X-100 for 10 min, blocked with 2% bovine serum albumin (BSA) for 2 h at RT, and then incubated with SREBP2 (ab112046, Abcam Plc., UK), HMGCR (ab174830, Abcam) and CYP7A1 (EL907377–100, EterLife) primary antibody respectively overnight at 4 °C. After removal of the primary antibody by washing with PBS, the sections were incubated with biotin conjugated goat anti-rabbit IgG for 15 min at RT. After washing with PBS, the sections were incubated in an HRP-conjugated avidin solution for 20 min followed by adding the developing solution. The images of cross sections were viewed and photographed by Axio Imager D2 microscope (Zeiss, Oberkochen, Germany).

### Cell culture and cell viability assay

HepG2 cells (SCSP-510) was purchased from the Chinese Cell Culture Center (Shanghai, China) and cultured in Eagle’s minimal essential medium (MEM), with 10% fetal bovine serum (FBS) and 1% penicillin and streptomycin and 1% non-essential amino acids (NEAA) and incubated at 37 °C in a humidified atmosphere of 5% CO2.

To determine the cell viability, HepG2 cells were seeded at 5 × 10^3^ cells/well into 96-well plates for 24 h, treated with quercetin at indicated concentrations (1, 5, 10, 30, 60, 90, 150, and 180 μmol l^− 1^) for 24 h and re-incubated with MTT solution at 37 °C for 4 h. Then the medium was removed and added 150 μl DMSO to each well, oscillated for 10 min. The absorbance was measured at 490 nm.

### Analysis of cholesterol in HepG2 cells

A cholesterol accumulation model of HepG2 cells caused by LDL was established. 2 × 10^5^ cells/well were seeded in 48-well plates. After 24 h, the cells were incubated without FBS for 1 h, and then were treated without (control) or with 25 μg ml^− 1^ LDL. The final concentrations of quercetin (Que; 30, 10 and 5 μmol l^− 1^) and LDL + Que. (quercetin 30, 10 μmol l^− 1^) were used as the treatment group. Hepatocytes were harvested for analysis of TC and protein.

### Real-time quantitative PCR analysis

RNA isolation, cDNA synthesis and real-time PCR analysis were performed as described previously [22]. Both human and rat gene primer sequences used for real-time PCR were shown in Table 1. Results were presented as levels of expression relative to those of controls after normalization to GADPH using the 2^-△△CT^ methods. Analysis was carried out in triplicates.

**Table 1:** Primer sequences used for real-time PCR analysis

Gene	Forward	Reverse
h-SREBP2	AAGTCTGGCGTTCTGAGGAA	AGGTCCACCTCATTGTCCAC
h-HMGCR	CTTGTGTGTCCTTGGTATTAGAGCTT	GCTGAGCTGCCAAATTGGA
h-SR-BI	CTTAAGAACGTGCGCATCGA	TGCTTTTGTGCCTGAACTCC
h-LXRɑ	GGAGGTACAACCCTGGGAGT	AGCAATGAGCAAGGCAAACT
h-LXRβ	CCCCTTCTTCTTCACCCACT	CGACTGTGACTGTGACTCCT
h-β-actin	CTGGAACGGTGAAGGTGACA	AAGGGACTTCCTGTAACAATGCA
r-SREBP2	AGAAGGAGAAAGGCGGACAA	TCTCCTGGCGCAGTTTATGA
r-HMGCR	AGAGCTGGCTTGAAACACCTGA	TTTGGACTGGAGACGGATGTAGA
r-CYP7A1	TTGGAATAAGGAGAAGGAAAGC	GGAGTTTGTGATGAAATGGACA
r-miR-33a	CCTCATAAGCGGTGCATTGTA	TATGCTTGTTCTCGTCTCTGTGTC
r-GAPDH	GGTGAAGGTCGGTGTGAACG	CTCGCTCCTGGAAGATGGTG

SREBP2 sterol regulatory element binding protein 2; HMGCR HMG-CoA reductase; CYP7A1 cytochrome P450 family 7 subfamily A member 1; LXRα liver X receptor ɑ; LXRβ liver X receptor β; SR-BI scavenger receptor class B type 1; GAPDH glyceraldehyde-3-phosphate dehydrogenase; miR-33a microRNA-33a

### Determination of SREBP2 and HMGCR protein by immunofluorescent staining

HepG2 cells were seeded on cover slips in 24-well plates and treated with quercetin at indicated concentrations. After 24 h, the supernatant was removed and washed with PBS 3 times and then fixed with 4% paraformaldehyde for 30 min. After removal of paraformaldehyde by aspiration and washing with PBS, cells were blocked with 2% BSA for 2 h, and then incubated with SREBP2 or HMGCR antibodies overnight at 4 °C. Cells were re-incubated with the secondary antibody for 1 h at room temperature. After washing with PBS, the slips were stained with DAPI solution for nuclei. The slips were observed by using Axio Imager D2 (Zeiss, Oberkochen, Germany), and the images of cells were photographed.

### Western blot analysis

After treatment, total cellular proteins were extracted from cells followed by determination of SREBP2, HMGCR and β-actin (ab8227, Abcam) protein expression by Western blot as described [26]. Bands were visualized with a ChemiDoc MP Imaging System (Bio-Rad, Hercules, CA, USA), and the density were quantitated based on three repeated experiments.

### Statistical analysis

All experiments were repeated three times. The results were analyzed using the SPSS 22.0 statistical software. The values were expressed as the mean ± SEM. Sample comparison was evaluated by one-way ANOVA and LSD or Dunnett’s T3 multiple comparison tests, and a *p*-value < 0.05 was considered statistically significant.

## Results

### MLF inhibited hepatic lipid accumulation in OA diet induced rats

**Fig. 1:**
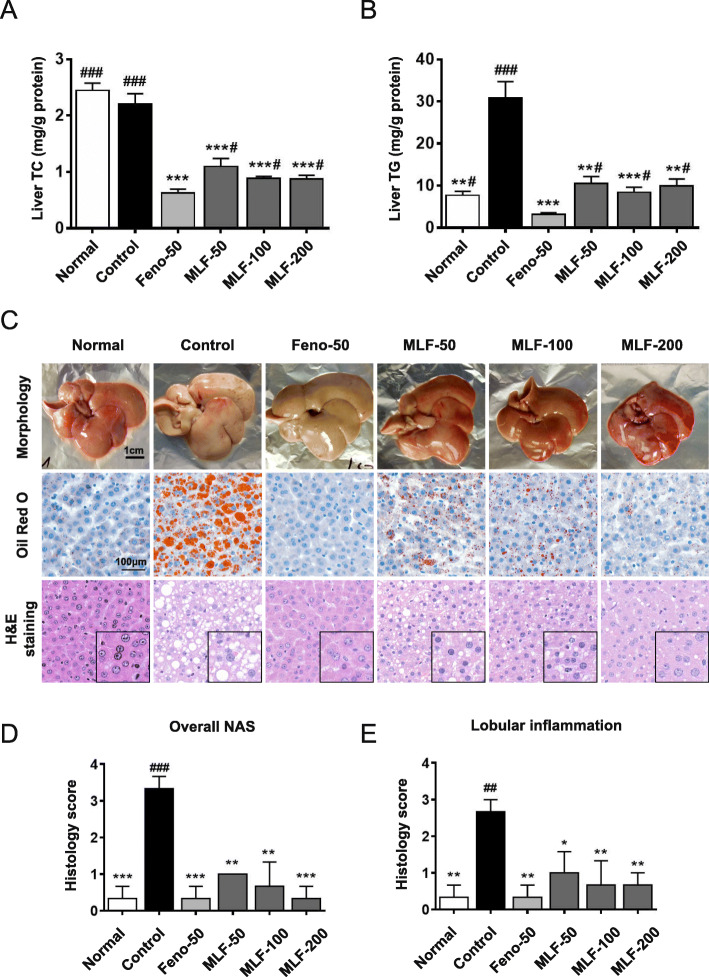
Regulation effects of MLF on hepatic lipid accumulation in NAFLD rats. **a** and **b** TG and TC levels in the liver with or without oral administration of MLF at 200, 100 and 50 mg kg^− 1^ d^− 1^. **c** Morphology, hematoxylin-eosin (H&E) staining and oil red O staining of liver tissues. Pictures were captured under a microscope (400×). **d** and **e.** H&E-stained liver sections were assessed for the NAFLD activity score (NAS) and lobular inflammation. Normal: normal group; Control: control group; Feno-50: positive control group (50 mg kg^− 1^); MLF: mulberry leaves extract in different dosage groups. Data were presented as mean ± SEM. *n* = 10, *p < 0.05, **p < 0.01, ***p < 0.001 vs. control group, #*p* < 0.05, ##*p* < 0.01, ###*p* < 0.001 vs. Feno-50 group

We initially determined the hepatic lipid content and found that TG level was significantly increased in OA fed control rats. MLF and fenofibrate treatment could significantly decrease the TG and TC levels in the liver (Fig. 1a and b).

To further determine the role of MLF, we assessed the hepatic histological and morphological changes by oil red O and H&E staining. Figure 1c demonstrated that there was significant hepatocyte hypertrophy and vacuolization with obvious hepatocyte damage caused by excessive lipid accumulation. However, fenofibrate and MLF treatment could reduce lipid droplets accumulation and alleviated the histological lesion in rat liver fed with OA. Notedly, the liver tissue morphology of the MLF-200 group was similar to that of Feno-50 group (Fig. 1c).

### MLF improved hepatic cholesterol metabolism in OA diet induced rats

**Fig. 2:**
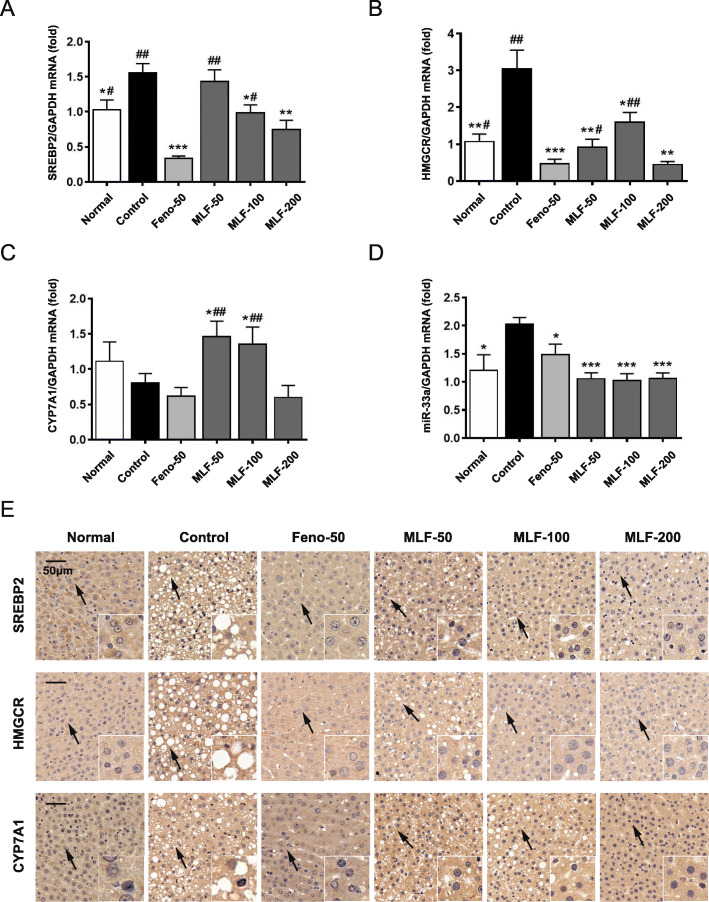
The effect on the regulation of cholesterol disorders of MLF. **a-d** Expression of SREBP2, HMGCR, CYP7A1, miR-33a mRNA was analyzed by quantitative real-time PCR. **e** Expression of SREBP2, HMGCR, CYP7A1 protein were analyzed by immunohistochemistry assay. Normal: normal group; Control: control group; Feno-50: positive control group (50 mg kg^− 1^); MLF: mulberry leaves extract in different dosage groups. Data were presented as mean ± SEM. n = 10, *p < 0.05, **p < 0.01, ***p < 0.001 vs. control group, #*p* < 0.05, ##*p* < 0.01, ###*p* < 0.001 vs. Feno-50 group

To further illustrate the potential mechanism of MLF on cholesterol metabolism, we assessed the expression levels of SREBP2, HMGCR and CYP7A1. As is shown in Fig. 2a and b, the hepatic SREBP2 and HMGCR expression were significantly induced in OA fed rats. Treatment of MLF and fenofibrate could decrease the expression of SREBP2 and HMGCR. Furthermore, treatment of MLF at different concentrations markedly up-regulated CYP7A1 mRNA expression, while down-regulated its inhibitor-miR-33a (Fig. 2c and d). In addition, similar results were obtained by immunohistochemistry assay, suggesting MLF could also regulate cholesterol metabolism genes in protein level (Fig. 2e).

### Quercetin inhibited hepatic cholesterol accumulation via regulating SREBP2 and HMGCR expression in HepG2 cells

**Fig. 3:**
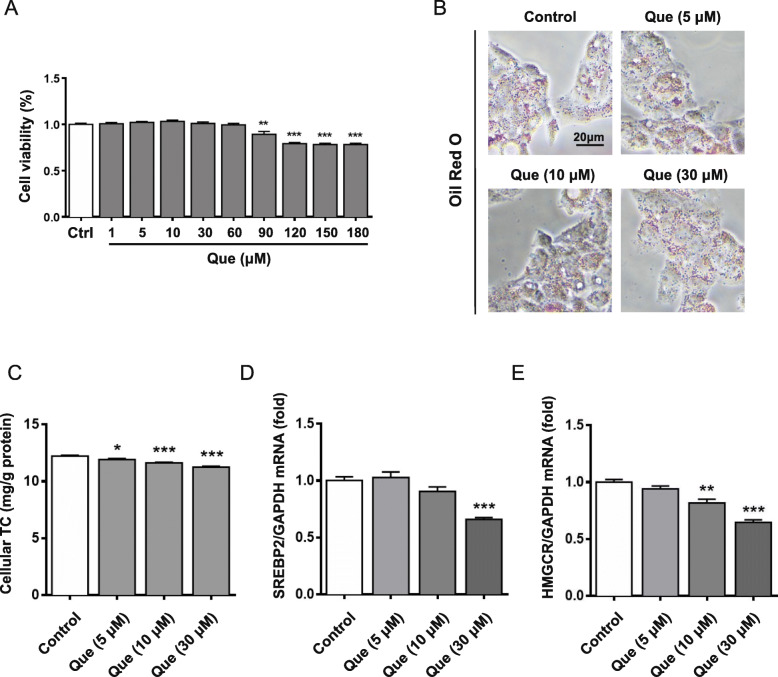
Results of modulating cholesterol level and lipid accumulation by quercetin in HepG2 cells. **a** Effects of quercetin on the viability of HepG2 cells. **b** Oil red O staining results with or without administration of quercetin after 24 h. **c** Cellular TC levels at 12 h. **d** and **e** mRNA expression of SREBP2 and HMGCR were quantified by quantitative real-time PCR. Control: control group; Que.: quercetin in different dosage groups (30, 10, 5 μmol l^− 1^). Data were presented as mean ± SEM. *n* = 6, *p < 0.05, **p < 0.01, ***p < 0.001 vs. control group

To further explore the mechanism of quercetin in regulating cholesterol metabolism, HepG2 cells were treated with quercetin at different concentrations. It was found that quercetin had no inhibitory effect on HepG2 cells activity at a concentration of 1–60 μM (Fig. 3a). As Fig. 3b showed, quercetin treatment could significantly reduce the lipid droplets accumulation in HepG2 cells, especially at dose of 30 μM. Consistently, total cholesterol also decreased with concentration increasing, and most significantly at 12 h (Fig. 3c).

**Fig. 4:**
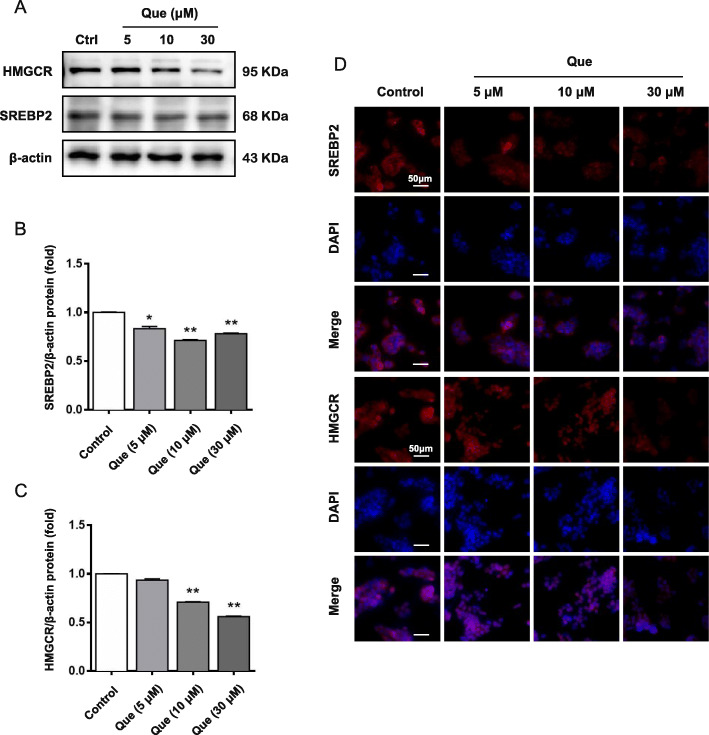
The effect on regulating cholesterol disorders and mechanism of quercetin on the HepG2 cells. **a-c** Expression of SREBP2 and HMGCR protein were analyzed by western blotting. **d** Expression of SREBP2, HMGCR protein were analyzed by immunofluorescence assay. Control: control group; Que.: quercetin in different dosage groups (30, 10, 5 μmol l^− 1^). Data were presented as mean ± SEM. n = 6, *p < 0.05, **p < 0.01 vs. control group

Next, the expression levels of SREBP2 and HMGCR were detected. As shown in Fig. 3d-e and Fig. 4a-c, the mRNA and protein levels of SREBP2 and HMGCR were decreased by quercetin. In addition, the inhibitory effect of SREBP2 and HMGCR expression by quercetin was further confirmed by immunofluorescent staining (Fig. 4d). These results imply that the cholesterol accumulation inhibition role of quercetin is related to reduction of cholesterol synthesis, particularly through regulating SREBP2 and HMGCR expression.

### Quercetin inhibited LDL induced cholesterol accumulation in HepG2 cells

**Fig. 5:**
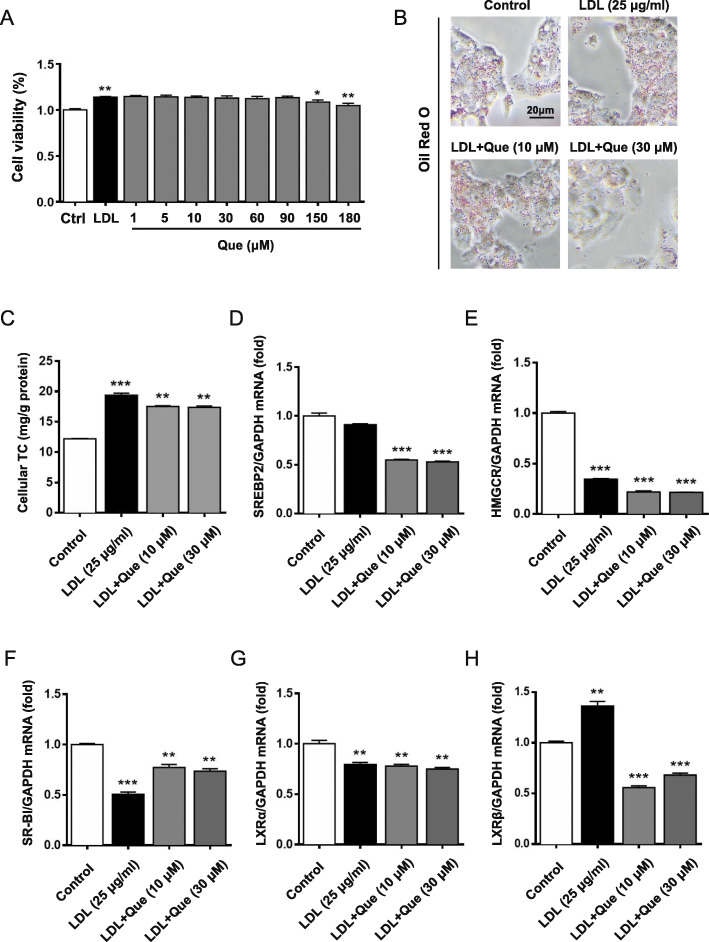
Results of modulating cholesterol level and lipid accumulation by quercetin in LDL-induced cholesterol laden HepG2 cells. **a** Effects of quercetin on the viability of HepG2 cells in LDL model. **b** Oil red O staining results with or without administration of quercetin after 24 h. **c** Cellular TC levels at 12 h. **d-h** mRNA expression of SREBP2, HMGCR, CYP7A1, SR-BI, LXRα and LXRβ. Control: control group; LDL: a high cholesterol level simulation model of HepG2 cells by low density lipoprotein (25 μg ml^− 1^); LDL + Que.: quercetin with LDL model HepG2 cells (30, 10 μmol l^− 1^). Data were presented as mean ± SEM. n = 6, *p < 0.05, **p < 0.01, ***p < 0.001 vs. control group

Quercetin is reported to be the major metabolite of mulberry flavonoids after intestinal flora and hepatic metabolism in vivo [18]. Therefore, to further define the role of MLF, the effect of quercetin on LDL-induced cholesterol accumulation was tested in HepG2 cells. As shown in Fig. 5a, quercetin had no inhibitory effect on cells viability at concentration 1–90 μM. The oil red O staining results showed that quercetin treatment could significantly inhibit LDL-induced lipid accumulation in HepG2 cells and gradually decrease the lipid droplets as the dose increased (Fig. 5b). Furthermore, we analyzed the total cholesterol levels in LDL-induced HepG2 cells after quercetin treatment. The results showed that after LDL induction, TC content was significantly stimulated, while the TC level of HepG2 cells decreased most significantly after the intervention of quercetin at 12 h (Fig. 5c).

### Quercetin regulated hepatic lipid related genes expression in LDL-induced cholesterol laden HepG2 cells

**Fig. 6:**
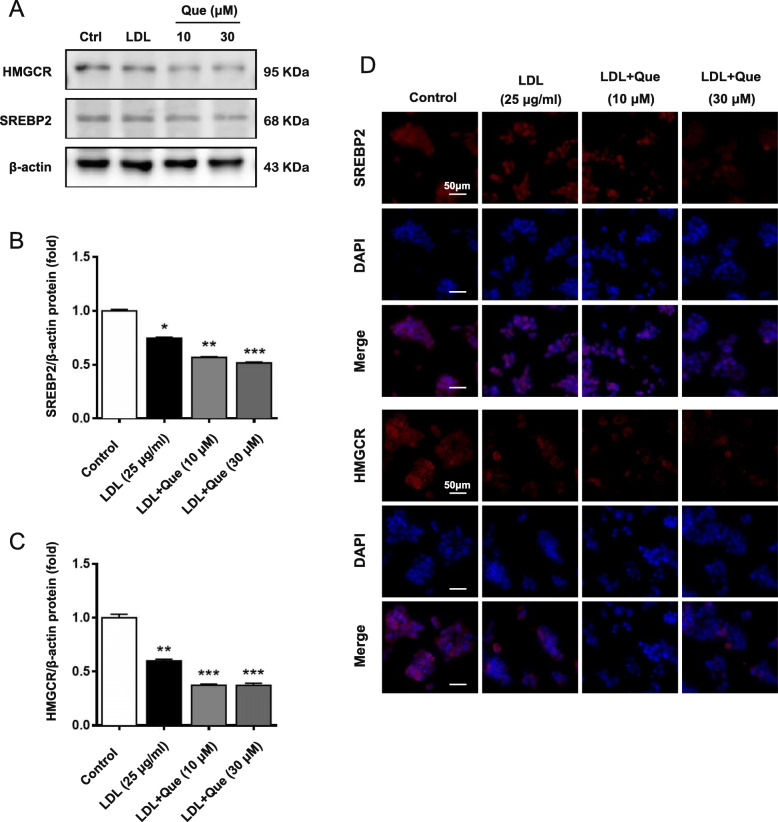
The effect on regulating cholesterol disorders and mechanism of quercetin on the HepG2 cells induced by LDL for 24 h. **a-c** Expression of SREBP2 and HMGCR protein were analyzed by western blotting. **d** Expression of SREBP2 and HMGCR protein were analyzed by immunofluorescence assay. Control: control group; LDL: a high cholesterol level simulation model of HepG2 cells by low density lipoprotein (25 μg ml^− 1^); LDL + Que.: quercetin with LDL model HepG2 cells (30, 10 μmol l^− 1^). Data were presented as mean ± SEM. *n* = 6, *p < 0.05, **p < 0.01, ***p < 0.001 vs. control group

To further determine the potential mechanism of quercetin in cholesterol metabolism, cells were treated with LDL or LDL with quercetin at different concentrations. The results showed that quercetin could significantly down-regulate the mRNA expression of SREBP2 and HMGCR (Fig. 5d and e). In addition, scavenger receptor class B type 1 (SR-BI) mRNA expression was induced by quercetin treatment (Fig. 5f). Interestingly, quercetin could significantly decrease the mRNA expression of LXRβ in LDL-induced HepG2 cells, while had no significant effect on LXRα (Fig. 5g and h). Consistent with the mRNA results, quercetin treatment could markedly reduce the SREBP2 and HMGCR protein expression in LDL induced cells (Fig. 6a-6d).

## Discussion

NAFLD is a most common metabolic disease, which can gradually lead to series of disease, including liver cancer and cardiovascular disease. Initial stage of NAFLD is mild and often developing slowly [5]. Clinic research indicated that early treatment or life-style modification significantly reduced the incidence of complications. Although the causes of NAFLD is multi-factorial and not entirely clarified, excessive TG and cholesterol induced pathological changes in liver is considered as an important risk factor on NAFLD occurrence and development [27].

Under the state of NAFLD, major pathological changes are excessive lipid accumulation induced liver steatosis, tissue damage and necrosis [9]. The major pathophysiological changes include up-regulation of SREBP2, HMGCR, down-regulation of CYP7A1. SREBP2 and HMGCR are closely related to cholesterol synthesis in mammal liver. Furthermore, nearly 40% of cholesterol is converted to bile acids by CYP7A1. miR-33a is involved in the regulation of metabolic disorders, and specifically inhibits CYP7A1 mRNA [28]. These changes resulted in increasing of hepatic cholesterol content, decreasing conversion rate of cholesterol into bile acids, reducing uptake rate of cholesterol from the serum. Finally, it could cause destruction of cholesterol homeostasis and increasing the risk of CVD [29].

Currently, there are no specific drugs approved for NAFLD treatment. Cholesterol-lowering drugs, such as statins, have been used to treat NAFLD in clinic. However, there are also some inevitable side effects [30]. Recently, dietary supplements are becoming an option for the prevention and treatment of NAFLD. As a medicinal and edible plants resource, MLF has been proved to be useful for preventing the development of NAFLD [31]. Recent studies have confirmed that treatment with MLF for 4 weeks can improve the lipid status of patients with hypercholesterolemia, decrease serum TG, LDL and TC/ HDL ratios, and normalize the serum LDL level [32]. While the mechanism studies are still lacking, which limit its clinic use.

As previously reported [14], OA can induce NAFLD via promote fat deposition in the liver. In this study, we investigated the regulatory mechanism of MLF on cholesterol metabolism in OA-induced rats. It implied that the prevention of NAFLD by MLF in HFD induced rats may be attributed to inhibiting excessive production of cholesterol. Our results showed that MLF could significantly reduce the TC level accompanied by the up-regulation of CYP7A1 expression in liver. In addition, we found that the high dose MLF (200 mg/kg) showed comparable lipid-lower effect of fenofibrate without causing adverse effect in this rat model.

The biosynthesis of cholesterol begins with taking acetyl-coenzyme A as the raw material and generates mevalonic acid pathway through the action of HMGCR and other enzymes. It is the speed limit and irreversible step of cholesterol synthesis. Therefore, HMGCR is of great medical significance which is strictly regulated by the nuclear transcription factor SREBP2. In our results, the expression level of SREBP2 and HMGCR were down-regulated, which inhibited the biosynthesis of cholesterol and further reduced the cholesterol accumulation in liver. Similarly, previous reports have shown that MLF significantly reduced blood glucose, serum TC, LDL-c and TG, and inhibit excessive accumulation of hepatic lipids in mice induced by high-fat diet [33, 34].

Quercetin, as one of the main flavonoids in mulberry leaves, is reported to be the major metabolite of mulberry flavonoids in vivo [18]. it exerts the functions of modulating cholesterol level and anti-inflammation [35, 36]. By using LDL induced cholesterol loading HepG2 cell model, we found that quercetin could reduce LDL induced cholesterol accumulation. Mechanistically, both mRNA and protein expression level of SREBP2 and HMGCR were down-regulated by quercetin, resulted in the regulation of cholesterol metabolism disorders. In addition, it also indicated that quercetin could inhibit cholesterol accumulation, rather than increase TG accumulation in HepG2 cells by up-regulating LXRβ. Therefore, quercetin can assist HepG2 cells to remove LDL from the circulation. Although recent study suggested that the role in treating NAFLD has been partially elucidated as the regulation of lipid metabolism related SREBP-1c, FAS and SCD1 [14], the mechanism for regulating TC metabolism are still unclear, we speculate that it may be related to the synthesis and secretion of cholesterol regulated by SREBP2 and HMGCR.

Therefore, we have proved that MLF and quercetin can inhibit the de novo synthesis of cholesterol and promote the elimination of cholesterol, thus preventing the accumulation of cholesterol in the liver. In addition, through the decrease of LXRβ mRNA expression, it is reasonable to speculate that quercetin can inhibit the expression of lipid-related mRNA and protein in the liver lipid metabolism pathway, but this needs further study.

## Conclusion

Our study demonstrated that MLF and quercetin could reduce the excessive cholesterol accumulation in vivo and in vitro. The cholesterol-lowering effect of MLF might attribute to its role in down-regulating the expression of SREBP2 and HMGCR, and up-regulating the expression of CYP7A1. These results suggest that quercetin, as the main metabolite of MLF, can regulate cholesterol metabolic disorders and reduce the risk of NAFLD.

## Supplementary information

**Additional file 1: Supplementary Fig. 1.** Full scans of western-blot data were shown in Fig. 4. Rectangles delimit cropped areas used in the indicated panels in Fig. 4. **Supplementary Fig. 2.** Full scans of western-blot data were shown in Fig. 6. Rectangles delimit cropped areas used in the indicated panels in Fig. 6.

## Data Availability

Materials used and data collected in this study are available from the corresponding author on reasonable request.

## Abbreviations

BSA: bovine serum albumin
cDNA: complemental deoxyribonucleic acid
CVD: cardiovascular disease
CYP7A1: cholesterol-7α-hydroxylase
DAPI: 4′,6-diamidino-2-phenylindole
DMSO: dimethyl sulfoxide
FBS: fetal bovine serum
GAPDH: glyceraldehyde-3-phosphate dehydrogenase
H&E: hematoxylin-eosin
HMGCR: HMG-CoA reductase
HPLC: high performance liquid chromatography
LDL: low density lipoprotein
LDL-C: low density lipoprotein cholesterol
LXRɑ: liver X receptor ɑ
LXRβ: liver X receptor β
MEM: Eagle’s minimal essential medium
miRNA: microRNA
MLF: mulberry leaves flavonoids
NAFLD: nonalcoholic fatty liver disease
NAS: NAFLD activity score
NEAA: non-essential amino acids
OA: orotic acid
PBS: phosphate buffer saline
RT: room temperature
SR-BI: scavenger receptor class B type 1
SREBP2: sterol regulatory element binding protein 2
TC: total cholesterol
TG: triglyceride
