# Supplementary material for: Regulation effects of total flavonoids in Morus alba L. on hepatic cholesterol disorders in orotic acid induced NAFLD rats
Source: BMC Complement Med Ther. 2020 Aug 17;20:257. doi: 10.1186/s12906-020-03052-w (PMC7433163; doi:10.1186/s12906-020-03052-w)

**Supplementary materials**
**For**
**Regulation effects of total flavonoids in *Morus alba* L. on hepatic cholesterol disorders in orotic acid induced NAFLD rats**

Yucheng Hu^1†^, Jingqi Xu^2†^, Qian Chen^1^, Mengyang Liu^2^, Sijian Wang^1^, Haiyang Yu^1^, Yi Zhang^2*^ and Tao Wang^1*^

**Figure Legend for Supplementary Data**

**Supplementary Figure 1. Full scans of western-blot data were shown in Figure 4.** Rectangles delimit cropped areas used in the indicated panels in Figure 4.

**Supplementary Figure 2. Full scans of western-blot data were shown in Figure 6.** Rectangles delimit cropped areas used in the indicated panels in Figure 6.

**Supplementary Figure 1**

Figure 4A


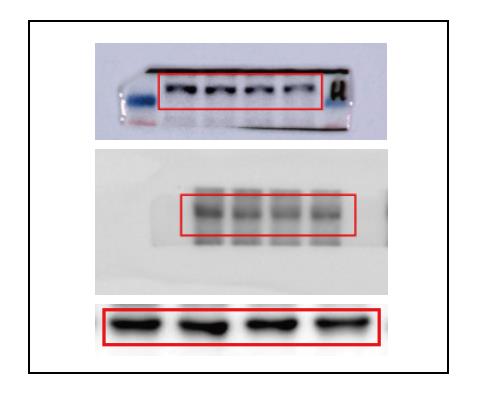


**Supplementary Figure 2**

Figure 6A


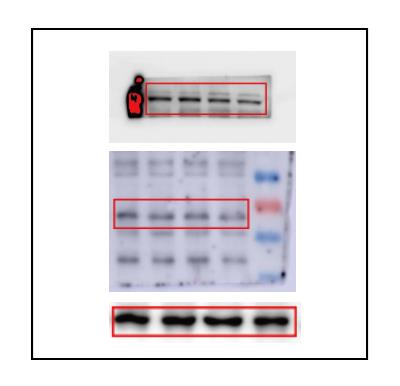

Supplement: Supplementary file 1 — Additional file 1: Supplementary Fig. 1. Full scans of western-blot data were shown in Fig. 4. Rectangles delimit cropped areas used in the indicated panels in Fig. 4. Supplementary Fig. 2. Full scans of western-blot data were shown in Fig. 6. Rectangles delimit cropped areas used in the indicated panels in Fig. 6. [file 12906_2020_3052_MOESM1_ESM.docx]
